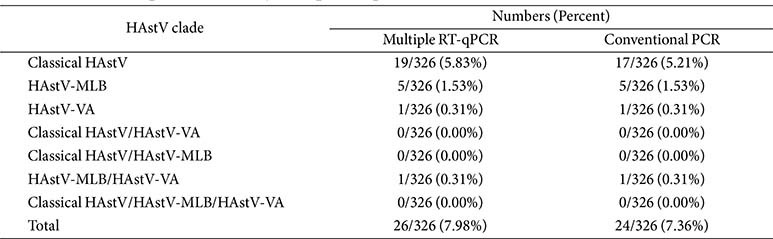# Development of a Multiplex TaqMan Real-Time RT-PCR Assay for the Rapid Differential Detection of Classic, MLB-Clade and VA-Clade Human Astroviruses

**DOI:** 10.4014/jmb.2506.06008

**Published:** 2025-09-22

**Authors:** Peipei Zheng, Jianhang Wang, Hongwei Yu, Qingping Luo, Huijie Gao, Zengjun Jin, Yilei Shi, Hanzhong Wang, Penghui Li, Feng Tian

**Affiliations:** 1 School of Medicine, Hebei University of Engineering, Handan 056038, P.R. China; 2 Hebei Key Laboratory of Medical Data Science, Handan, Hebei Province 056038, P.R. China; 3 College of Life Science and Food Engineering, Hebei University of Engineering, Handan 056038, P.R. China; 4 Key Laboratory of Prevention and Control Agents for Animal Bacteriosis (Ministry of Agriculture and Rural Affairs), Institute of Animal Husbandry and Veterinary, Hubei Academy of Agricultural Sciences, Special One, Nanhuyaoyuan, Hongshan District, Wuhan 430064, P.R. China; 5 The Affiliated Hospital, Hebei Engineering University, Handan 056029, P.R. China; 6 CAS Key Laboratory of Special Pathogens and Biosafety, Wuhan Institute of Virology, Center for Biosafety Mega- Science, Chinese Academy of Sciences, Wuhan 430071, P.R. China

**Keywords:** Human astrovirus, multiplex TaqMan real-time RT-PCR, clade, nucleic acid detection

## Abstract

The classical human astrovirus (HAstV) causes acute or severe gastroenteritis mainly among children and the elderly populations. The novel divergent HAstV-VA and HAstV-MLB clades related to encephalitis in immunocompromised patients, are genetically more similar to certain animal astroviruses, and have the potential for cross-species transmission. To identify all the three HAstV clades in a single reaction, we developed a quantitative reverse transcription PCR (RT-qPCR) method. Primers and probes were designed based on the conserved regions of the HAstV genomes that exhibited inter-clade divergence. This method was highly sensitive, as the detection limits were 95, 12.5 and 25 copies/μl for the classical HAstV, HAstV-MLB and HAstV-VA strains, respectively. Intra- and inter-assay variability revealed excellent reproducibility. Furthermore, this multiplex assay showed no cross-reactivity with other human pathogens. A total of 326 anal swabs from outpatients with AGE were examined: 5.83% were positive for the classical HAstV, 1.53% for the HAstV-MLB, and 0.31% for the HAstV-VA, while 0.31% for co-infections of HAstV-MLB/HAstV-VA. Conclusively, the developed multiplex RT-qPCR assay represents a tool with potential for laboratory and clinical diagnoses, epidemiological surveillance, prevention and control of the HAstV infection.

## Introduction

The human astrovirus (HAstV), initially identified in 1975, is a non-enveloped, positive-sense, single-stranded RNA virus, classified within the family *Astroviridae* [1-3]. The initially recognized HAstV strains were classified into eight serotypes, currently referred to as canonical or classical HAstV (HAstV1–8) subtypes [4, 5]. Infections caused by classical HAstV strains commonly lead to acute or severe gastroenteritis, primarily in children and the elderly [3, 6]. In recent years, two new and highly divergent clades of HAstV—HAstV-MLB and HAstV-VA—have been identified in fecal samples; notably, these new strains exhibit the capacity to spread systemically in patients with compromised immune system, leading to fatal meningitis and encephalitis [1, 3]. Phylogenetic analyses indicate that these divergent MLB and VA clades are genetically highly similar to bovine, porcine, mink, and ovine astroviruses compared to the classical HAstV. Therefore, these new clades are referred to as either ‘atypical’ or ‘animal-like’ strains, of probable zoonotic origin, suggesting the occurrence of animal–human host crossover events [3, 6, 7].

The viral genome is a single-stranded, positive-sense RNA measuring approximately 6.8 kb in length, comprising three key overlapping open reading frames (ORFs) [6, 8, 9]. Notably, the ORF1a and ORF1b encode for non-structural proteins, namely the serine protease and the RNA-dependent RNA polymerase (RdRp), respectively [4, 8, 10]. The ORF2 encodes viral capsid protein that forms the outer shell of the viral particles [11, 12]. Notably, the length of ORFs varies among the HAstV subtypes [13]. Additionally, research has identified a fourth ORF, designated as ORFX, in some astrovirus strains, including the classical HAstV strains. The ORFX encodes a viroporin-like protein, which plays a crucial role in virus assembly, release, or both [8, 10, 12]. The HAstVs have been characterized by increasing infections rates, rapid recombination and mutation, substantial clinical features, along with the rising healthcare costs and economic burdens making a significant threat to public health [14-16].

To prevent and manage the spread of HAstV infections, research should prioritize the development of a HAstV vaccine. Nonetheless, currently there is lack of either effective vaccines or antivirals [17]. Owing to the increasing prevalence of the virus and the potential for cross-species transmission, identifying and monitoring of HAstVs during outbreaks remain crucial in the efforts to mitigate their spread within the affected communities [14, 16]. Commercially available ELISA kits—with the capacity to facilitate rapid and simultaneous analysis of large number of clinical specimens—have been used to detect HAstV antigens. However, when detecting viruses in clinical samples, this method is incapable of distinguishing different HAstV clades. The newly identified MLB and VA clades differ genetically in comparison to the classical HAstV that causes acute gastroenteritis (AGE). Notably, since 2008, these novel clades have been increasingly observed in fecal samples of children with gastroenteritis, and are associated with central nervous system, respiratory, and disseminated infections [12, 16, 18]. Co-infections involving the classical HAstV, HAstV-MLB, and HAstV-VA have been observed in clinical studies, indicating a potential opportunity for intra- and inter-genotype recombination between distinct clades of HAstV strains [14]. Thus, there is a critical need to develop a rapid and convenient method for molecular epidemiology and differential detection of the classical HAstV, HAstV-MLB and HAstV-VA strains. Various molecular techniques, including multiplex polymerase chain reaction (PCR) and real-time PCR, have been extensively used to identify the HAstV strains [19-22]. However, these methods exhibit significant limitation in simultaneously distinguishing the classical HAstV, HAstV-MLB and HAstV-VA strains in a single reaction. Currently, no research has developed a robust, convenient, and high-throughput molecular method that can simultaneously detect all three clades in a single reaction. Therefore, the development of a multiplex RT-qPCR assay to facilitate the typing of HAstV positive samples provides an ideal molecular assay for high-throughput monitoring of the kinetics of these increasingly prevalent HAstV strains. Additionally, quantitative analysis using such a technique could offer information on the viral load in clinical specimens.

This study aimed to develop a highly sensitive RT-qPCR assay with the capability to simultaneously detect and identify all the three clades (classic, MLB, and VA) of HAstV in a single reaction, to facilitate simultaneous subgrouping and rapid detection of these strains.

## Methods

### Viruses, Bacteria and Samples Used

The following viruses and bacteria were obtained from our laboratory: Enterovirus 71 (EV71, GZCII-P30), Langat virus (LGTV, TP21), coxsackievirus B3 (CVB3, AH30), influenza A hemagglutinin type 1 and neuraminidase type 1 (H1N1) virus (A/PR/8/34), Zika virus (ZIKV, SZ-WIV01), dengue virus (DENV, TSVO1), Japanese encephalitis virus (JEV, SA 14-14-2), *Escherichia coli* (*E. coli*, ATCC25922), *Campylobacter jejuni* (*C. jejuni*, NCTC11168), *Campylobacter coli* (*C. coli*, ATCC43478), and *Salmonella* (CVCC 540). A total of 326 anal swabs were collected from outpatients with AGE at the Affiliated Hospital of Hebei Engineering University, from 2023 to 2024. This study received ethical approval from the Biomedical Ethics Committee of School of Medicine, Hebei University of Engineering (Approval No. BER-YXY-2024028), and was performed in accordance with the principles of the Declaration of Helsinki. The data used in this study were anonymized to enhance privacy and confidentiality.

### Design of Primers and Probes

Nucleotide sequences of the classical HAstV, HAstV-MLB, and HAstV-VA genomes were retrieved from the NCBI nucleotide database. A total of 30 representative strains of the three HAstV clades were selected based on genetic variability and location of specimen collection. These strains were then aligned to identify conserved regions that exhibited inter-clade divergence using the DNAMAN software version 6.0.3.40 (Lynnon Biosoft, USA). Based on the results from the HAstV sequence alignment (Fig. S1), we developed three sets of primers and three sets of probes (Table 1) from the most divergent, yet conserved region specific to each HAstV clade using the Primer Premier 5 software (Premier, Canada). To prevent interference among fluorescence signals in this multiplex system, FAM (Carboxy Fluorescein), Rhodamine X (ROX), and Cyanine5 (CY5) were selected to label the classical HAstV, HAstV-MLB, and HAstV-VA probes, respectively. All primers and probes were constructed by the Tsingke Biotech Co. Ltd., (China).

### Preparation of Standard Templates

To evaluate the analytical sensitivity of the multiplex assay, recombinant standard plasmids for the classical HAstV, HAstV-MLB, and HAstV-VA strains were constructed based on the following method. The JZ strain (classical HAstV), SY071 strain (HAstV-MLB) and ITA/2018/205.18-5 strain (HAstV-VA) synthesized by the Tsingke Biotech Co. Ltd., served as template for the amplification of target gene. The partial RdRp-capsid gene fragment was amplified using PCR based on the classical HAstV, HAstV-MLB, and HAstV-VA RdRp-VP1 primers. The PCR products were purified using the agarose gel DNA fragment recovery kit (TIANGEN biotech Co., Ltd., China) according to the recommended protocol, then cloned into the pUC57 vector, and subsequently transformed into *E. coli* DH5α. Following blue-white screening, the positive colonies were verified using direct DNA sequencing by the Tsingke Biotech Co., Ltd. The plasmids were extracted using endotoxin-free plasmid extraction kit (Tiangen Biotech Co., Ltd.). The concentration of the standard plasmid was determined using the NanoDrop One/OneC spectrophotometer (Thermo Fisher Scientific, USA), and the copy number (copies/μl) was calculated using the following formula: 6.02 × 10^23^ copies/mol × concentration/average molecular weight [23]. The recombinant standard plasmids for the classical HAstV, HAstV-MLB and HAstV-VA were 10-fold diluted before use.

### Optimization of the Multiplex RT-qPCR

The multiplex RT-qPCR assay was performed using a 10 μl Probe qPCR Mix with UNG (Takara Bio, Japan), combined with all primers, templates, probes, and nuclease-free water to a final volume of 20 μl. The primer and probe concentrations for the classical HAstV, HAstV-MLB, and HAstV-VA strains were optimized to facilitate the concurrent detection of the classical HAstV, HAstV-MLB, and HAstV-VA strains with minimum quantification cycle (Cq) values. Additionally, the optimal annealing temperature of the multiplex qPCR was assessed between 50°C and 65°C, with a temperature gradient of 1°C. The optimal conditions were defined as those that facilitated the simultaneous detection of the three strains with minimum Cq values. All the RT-qPCR assays were conducted using QuantStudio Real-Time PCR (ABI, USA). The optimal qPCR system comprised the following: 10 μl Probe qPCR Mix with UNG, 2 μl templates, and 4.2 μl nuclease-free water, along with 0.4 μl primers (10 μM) for HAstV-classic-F / HAstV-classic-R, 0.4 μl primers (10 μM) for HAstV-MLB-F / HAstV-MLB-R, 0.4 μl primers (10 μM) for HAstV-VA-F / HAstV-VA-R, 0.8 μl primers (10 μM) for HAstV-classic-probe, 0.2 μl primers (10 μM) for HAstV-MLB-probe / HAstV-VA-probe. The optimal reaction conditions for the multiplex qPCR involved initial UNG action at 25°C for 10 min, 95°C hold for 30 s, followed by 40 cycles at 95°C for 5 s and 60°C for 30 s.

### Specificity of the Multiplex RT-qPCR

The specificity of the multiplex RT-qPCR was evaluated by assessing its reactivity with the cDNA derived from the classical HAstV, HAstV-MLB, and HAstV-VA, influenza A H1N1, ZIKV, EV71, DENV, CVB3, LGTV, JEV, and the DNA derived from the of *E. coli*, *C. jejuni*, *C. coli*, and *Salmonella*. Nuclease-free water served as a negative control.

### Sensitivity of the Multiplex RT-qPCR

To test the sensitivity of the multiplex qPCR, 10-fold dilutions for the classical HAstV, HAstV-MLB, and HAstV-VA—ranging from 1 × 10^8^ copies/μl to 1 × 10^0^ copies/μl—were prepared and used as templates. Nuclease-free water was used as a negative control. The results were compared to those obtained from the conventional PCR. The standard curves of the multiplex qPCR were constructed by plotting the Cq values against the logarithm of the initial standard copy numbers, subsequently, the correlation coefficient (R^2^) and amplification efficiency (Eff%) values were determined to evaluate the detection performance. Furthermore, the presumed limit of detection (LOD) was defined as the lowest concentration of the standard plasmids that could be reproducibly detected. The LOD was fine-tuned by testing the standard plasmids at the presumed LOD and its plus or minus two-serial dilutions [24].

### Repeatability of the Multiplex RT-qPCR

The assay was performed under optimized reaction conditions over three separate days, involving three replicates per reaction, utilizing 10-fold dilutions of standard plasmids ranging from 10^6^ to 10^4^ copies/μl. The intra- and inter-group coefficient of variation (CV) defined as the ratio of the standard deviation to the Cq mean for each dilutions, was determined to assess repeatability across all the concentrations.

### Verification of the Multiplex RT-qPCR

The existence of pathogens at low and undetectable concentrations is typically associated with misdiagnosis of co-infections. To mimic the occurrence of co-infections of the classical HAstV, HAstV-MLB, and HAstV-VA, combinations of the plasmid standards from the three pathogens at their LODs served as templates for the assay. The simulated co-infections of HAstVs were evaluated using the multiplex RT-qPCR assay. Finally, clinical samples were used to assess the clinical performance of the multiplex RT-qPCR assay. Anal swabs of outpatients with AGE were collected at the Affiliated Hospital of Hebei Engineering University, from week 46 in 2023 to week 2 in 2024. Viral RNAs were extracted, transcribed into cDNA, and screened for the classical HAstV, HAstV-MLB, and HAstV-VA using the developed multiplex RT-qPCR assay analysis and conventional PCR with the primers listed in Table 1 [21]. Subsequently, a comparison was conducted to assess the performance of the multiplex RT-qPCR and conventional PCR.

## Results

### Establishment of Standard Curve of the Multiplex RT-qPCR Assay

**Fig. 1:**
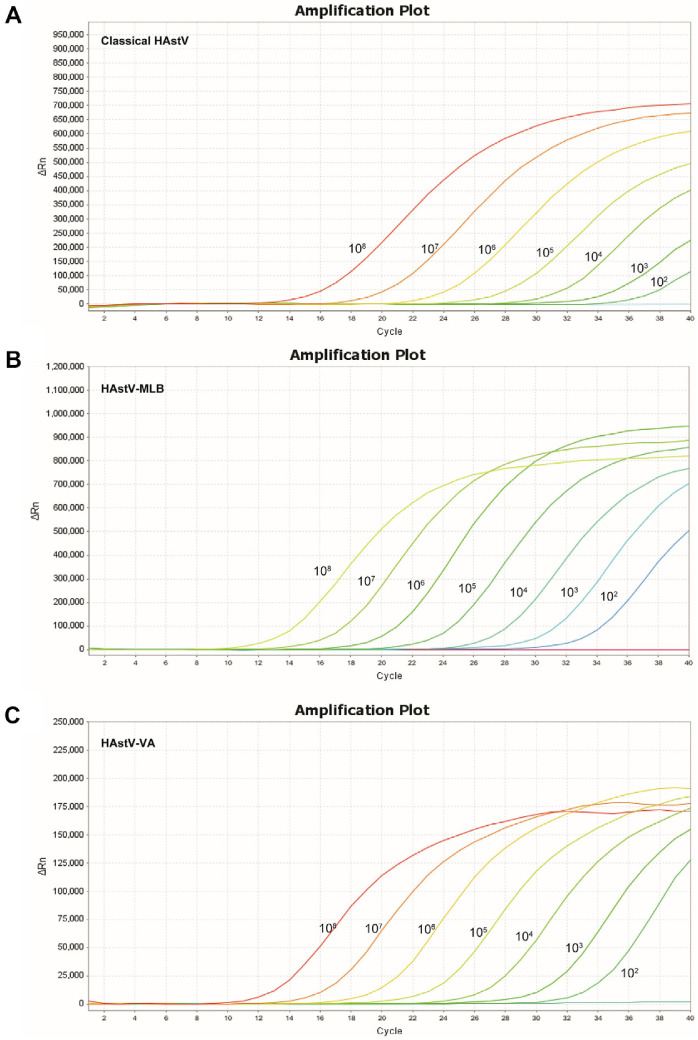
Amplification curves for the multiple RT-qPCR assay. (**A**) Amplification plot for the classical HAstV plasmid. (**B**) Amplification plot for the HAstV-MLB plasmid. (**C**) Amplification plot for the HAstV-VA plasmid.

**Fig. 2:**
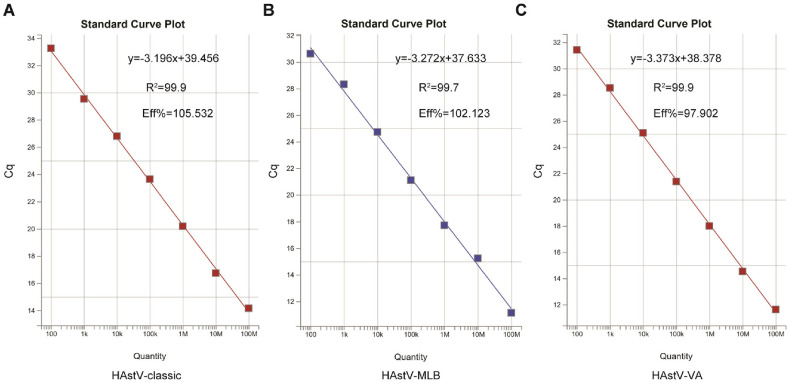
Standard curves of the multiple qPCR assay for detecting: ( A ) the classical HAstV, (B) HAstV-MLB, and (C) HAstV-VA.

To construct the standard curve of the multiplex qPCR, standard plasmids from the classical HAstV, HAstV-MLB and HAstV-VA clades were diluted 10-fold with deionized distilled water. Dilutions comprising 10^8^ to 10^2^ copies/μl were selected to conduct the multiplex real-time PCR detection under optimized conditions. As shown in Fig. 1, the amplification plots demonstrated strong correlation and consistency in the detection of individual target gene. The standard curves for the classical HAstV, HAstV-MLB, and HAstV-VA were constructed by plotting the copy number on the x-axis and Cq value on the y-axis. The Eff% values for detection of individual target gene were within the range of 98-106% (Fig. 2). The standard equation for the classical HAstV was y = -3.196x + 39.456, R^2^ = 0.999; for HAstV-MLB was y = -3.272x + 37.633, R^2^ = 0.997; and for HAstV-MLB was y = -3.373x + 38.378, R^2^ = 0.999 (Fig. 2).

### Specificity of the Multiplex RT-qPCR Assay

**Fig. 3:**
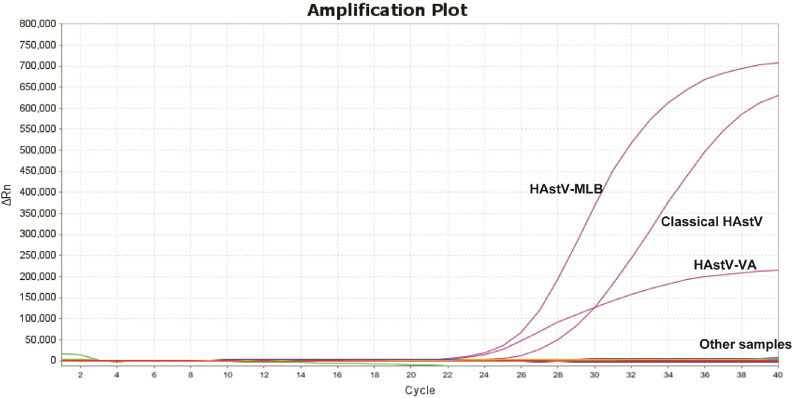
Specificity of the multiple RT-qPCR assay. Only the classical HAstV, HAstV-MLB and HAstV-VA strains produced three positive fluorescence signals, and no positive signals were observed with other human pathogens (EV71, DENV, CVB3, H1N1, LGTV, ZIKV, JEV, *C. jejuni*, *E. coli*, *C. coli*, and *Salmonella*) and nuclease-free water.

To evaluate the specificity of the multiplex RT-qPCR, the cDNA or DNA of the key pathogen prevalent in humans were detected as templates. Notably, only the classical HAstV, HAstV-MLB, and HAstV-VA strains produced three positive fluorescence signals, with no positive signals detected with other viruses (CVB3, H1N1, EV71, DENV, ZIKV, LGTV, and JEV), bacteria (*E. coli*, *C. jejuni*, *C. coli*, and *Salmonella*), and nuclease-free water (Fig. 3).

### Sensitivity of the Multiplex RT-qPCR Assay

**Fig. 4:**
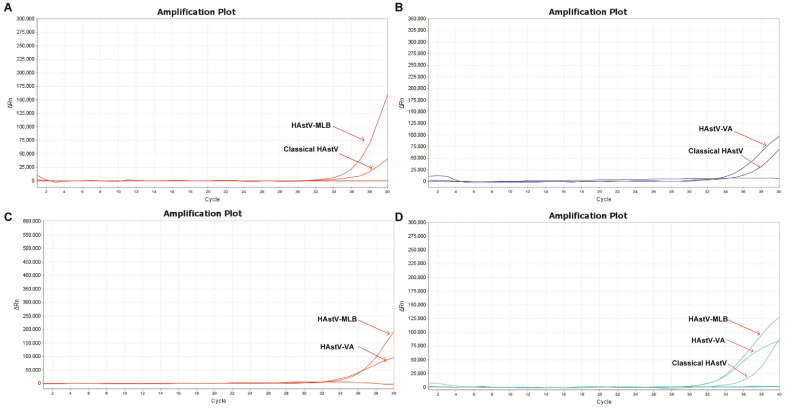
Coinfection simulation experiments with the classical HAstV, HAstV-MLB, and HAstV-VA clades at concentrations of the LOD.

To determine the sensitivity, 10-fold serial dilutions of standard plasmids were added to the amplification system. A Cq value in the 32-34 range demonstrated reliability for LOD concentrations, with values exceeding 35 indicating unreliability. Therefore, the positive detection threshold Cq value was set at 35. The multiplex qPCR showed high sensitivity in detecting the classical HAstV, HAstV-MLB, and HAstV-VA strains with LODs of 95, 12.5, and 25 copies/μl, respectively, which were about 100-fold higher compared to those obtained using the conventional PCR methods (Fig. S2). Moreover, all standard plasmids with concentrations at the LOD were utilized to conduct a comprehensive co-infection simulation. This approach exhibits advantage owing to its ability to detect not only mono-infection or duplex co-infections but also triplex co-infections, thereby enabling the detection of the classical HAstV, HAstV-MLB, and HAstV-VA, even at LOD concentrations within co-infection samples (Fig. 4).

### Repeatability of the Multiplex RT-qPCR Assay

Repeatability tests were conducted using three different known concentrations of standard plasmids. The results showed that the intra-assay CV ranged from 0.124 to 0.671% for the classical HAstV, 0.252 to 0.564% for the HAstV-MLB, and 0.146 to 0.505% for the HAstV-VA; additionally, the inter-assay CV ranged from 0.300 to 0.916%, 0.315 to 0.459%, and 0.086 to 0.638%, respectively. The CV of Cq was <1%, indicating high reproducibility for the established multiplex RT- qPCR method (Table 2).

### Evaluation Using the Clinical Samples

A total of 326 anal swabs were assessed using the multiplex RT-qPCR assay and conventional PCR. The positive detection rates of HAstVs for the multiplex RT-qPCR and conventional PCR were 7.98% (26/326) and 7.36% (24/326), respectively (Table 3). All samples that were positively detected using conventional PCR were also detected with the multiplex RT-qPCR (Table S1). The positive rates of detection for the mono-infection of either the HAstV-MLB, or HAstV-VA, and that of the co-infection of HAstV-MLB/HAstV-VA, were 1.53%, 0.31%, and 0.31%, respectively, as determined by the multiplex RT-qPCR, which were consistent with those of the conventional PCR. Additionally, 19 samples (5.83%) were detected as positive for the classical HAstV using the multiplex RT-qPCR; in contrast, 17 samples (5.21%) were detected as positive for the classical HAstV using the conventional PCR. This finding indicated that the multiplex qPCR was more sensitive compared to the conventional PCR. Co-infections with the classical HAstV/HAstV-MLB, the classical HAstV/HAstV-VA, and the classical HAstV/HAstV-MLB/HAstV-VA were not identified.

## Discussion

The HAstVs are highly contagious and represent the primary cause of viral diarrhea in children globally [5]. Emerging studies indicate that HAstVs are associated with fatal neurological diseases in immunocompromised patients [1, 2]. Currently, there are no vaccines or antivirals available against HAstVs [17], contributing to significant economic and health care burden in humans. Therefore, there is an urgent need to develop a reliable method to detect and distinguish the classical HAstV, HAstV-MLB and HAstV-VA strains, which could lay a foundation for the surveillance of these viruses, as well as the development of vaccines and antiviral therapeutic approaches.

Primer and probe design represent the most significant step in developing a successful multiplex RT-qPCR assay. After identifying conserved regions that exhibited inter-clade divergence, we designed primers and probes for the classical HAstV, HAstV-MLB and HAstV-VA using the RdRp-capsid gene. The three probes were selected from the conserved region of each of the HAstVs clades. We did not observe any cross reaction with other human pathogens using the BLAST search. Owing to the presence of distinct HAstV variants globally [25, 26], specific point mutations were detected in the classical HAstV, HAstV-MLB and HAstV-VA strains. To detect more diverse field HAstV strains, we introduced degenerate bases to the probes and primers as needed. The existence of secondary structures in primer/probe binding sites can substantially results in suboptimal amplification efficiency [27]. In this study, the Eff% values of each detection target were within the 98–106% range, and the results were in line with requirements [28, 29]. Additionally, three fluorescent dyes—including FAM, ROX, and CY5—were selected for the detection and typing of the classical HAstV, HAstV-MLB and HAstV-VA strains based on their different wavelengths. The characteristics of these three dyes ensured that all the three key clades were detected simultaneously in the same reaction tube without interference.

The sensitivity and specificity of the multiplex RT-qPCR are very crucial in ensuring effective detection. In oncology, accurate detection of low levels of HAstVs is essential for early diagnosis and prevention of HAstV infection in pediatric patients, especially given the potential for long-term viral excretion even after clinical symptoms have resolved [30]. It was generally accepted that the RT-qPCR was used as a surrogate for directly determining viral loads of human RNA viruses from fecal specimens, due to its fast turnaround times, high sensitivity and specificity, relatively low cost, and reduced risk of obtaining false-negatives [19, 31-35]. In contrast, despite their diagnostic significance, the conventional RT-PCR methods produced false-negative results in cases involving low viral loads (< 10^6^ RNA copies/ml) [36]. The LODs of the multiplex RT-qPCR in the single-tube assay system were determined as 95 copies/μl for the classical HAstV, 12.5 copies/μl for HAstV-MLB, and 25 copies/μl for HAstV-VA, which correspond to approximately 10^4^ copies/ml. This detection limit was substantially lower than the viral loads observed in patients with AGE, which ranged from 2.8 × 10^5^ copies/ml to 1.6 × 10^10^ copies/ml, with a median of 1.8 × 10^6^ copies/ml [37]. In the asymptomatic group, viral loads ranged from 3.1 × 10^6^ copies/ml to 1.6 × 10^11^ copies/ml, with a median of 5.8 × 10^9^ copies/ml [37]. Additionally, viral RNA concentrations in fecal specimens collected from children with AGE between days 1–3 ranged from 3.4 × 10^8^ to 1 × 10^13^ genomes/gram of fecal specimens [38], with a median of 1.2 × 10^10^ genomes/mg of fecal specimens [39]. Therefore, the LODs of our multiplex RT-qPCR assay were clinically adequate and sufficient for reliable detection of the HAstVs in actual patient specimens. Moreover, the specificity of the multiplex RT-qPCR was determined using different clades of HAstVs and various pathogens in humans. Notably, only the classical HAstV, HAstV-MLB, and HAstV-VA strains produced three positive fluorescence signals, while no positive signals were obtained with other viruses (H1N1, EV71, CVB3, ZIKV, DENV, LGTV, and JEV), bacteria (*C. jejuni*, *E. coli*, *C. coli*, and *Salmonella*), and nuclease-free water, indicating that the multiplex RT-qPCR had a high specificity for the detection of HAstV.

Co-infection in pediatric patients with AGE predicts a more severe clinical course [40]. Notably, HAstVs often co-infects with other pathogens, such as rotavirus, norovirus, and adenovirus [14-16, 19]. Additionally, co-infections could occur between different clades of HAstVs. Using nucleotide sequence analysis, we revealed a co-infection of HAstV1 and HMLB1 in the same patient [14]. Co-infection of multiple circulating lineages within the same host was conducive for recombination and emergence of novel AstVs within a species [41]. In this study, the multiplex co-infection of the classical HAstV, HAstV-MLB, and HAstV-VA was detected even at LOD concentrations, which demonstrated the effectiveness of this approach in simultaneous genogrouping of HAstVs.

In this study, the incidence of HAstV infection is estimated to range from 2% to 9% among children with AGE worldwide [42, 43]. Clinical samples analysis showed that 7.98% (26/326) of specimens were identified as HAstV in Handan city, which are results consistent with the reports for certain cities within China, such as 8.20% in Shanghai [44], 8.51% in Shandong [42], and 7.80% in Beijing [45]. In the present study, the classic HAstV remained the most prevalent strain in Handan city, with five cases of mono-infections of HAstV-MLB and one case of HAstV-VA, as well as one case of co-infection of HAstV-MLB/HAstV-VA. Although the co-infection rate of HAstVs was low, it provides an opportunity for the recombination of different clades of HAstVs and the emergence of novel HAstV strain [41]. Given the absence of the fragile lipid envelope, HAstVs harbor high environmental stability and remain infectious in feces, sewage, and other materials for longer periods even after shedding, thereby increasing the potential for different genotypes, clades, and serotypes to be involved in unique cross-species transmissions [25, 41, 46]. Therefore, there is an urgent need for developing a clinically rapid detection method for effectively controlling the spread of HAstV infections.

## Conclusion

Conclusively, we developed a multiplex RT-qPCR that detected and differentiated all the three clades of HAstV—namely, the classical HAstV, HAstV-MLB, and HAstV-VA—with high sensitivity, specificity, and reproducibility. This method demonstrated capacity to achieve rapid detection of the HAstV in a convenient and practical manner, suitable in laboratory settings, as well as in performing clinical diagnoses. Additionally, this approach is useful in the surveillance, prevention, and control of the HAstV infection.

## Supplemental Materials

Supplementary data for this paper are available on-line only at http://jmb.or.kr.



## Figures and Tables

**Table 1:** Primers and probes used in this study.

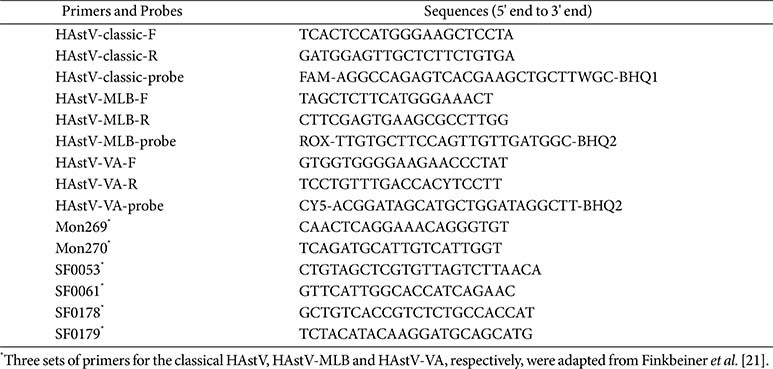

**Table 2:** Repeatability of the multiple RT-qPCR assay.

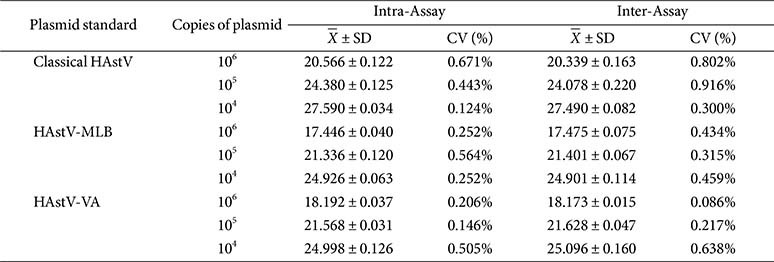

**Table 3:** Clinical samples examined by multiple RT-qPCR and conventional PCR.