# Supplementary material for: Development of a Multiplex TaqMan Real-Time RT-PCR Assay for the Rapid Differential Detection of Classic, MLB-Clade and VA-Clade Human Astroviruses
Source: J Microbiol Biotechnol. 2025 Sep 22;35:e2506008. doi: 10.4014/jmb.2506.06008 (PMC12535854; doi:10.4014/jmb.2506.06008)
Supplement: Supplementary file 1 [file jmb-35-e2506008-supple.pdf]

J. Microbiol. Biotechnol. 2025. 35: e2506008

https://doi.org/10.4014/jmb.2506.06008

Supplementary Figures and Tables

Fig. S1. Design of probes and primers for detecting (A) the classical HAstV, (B) HAstV-MLB, and (C) HAstV-VA. The primers are in the black box and the probes are in the red box.

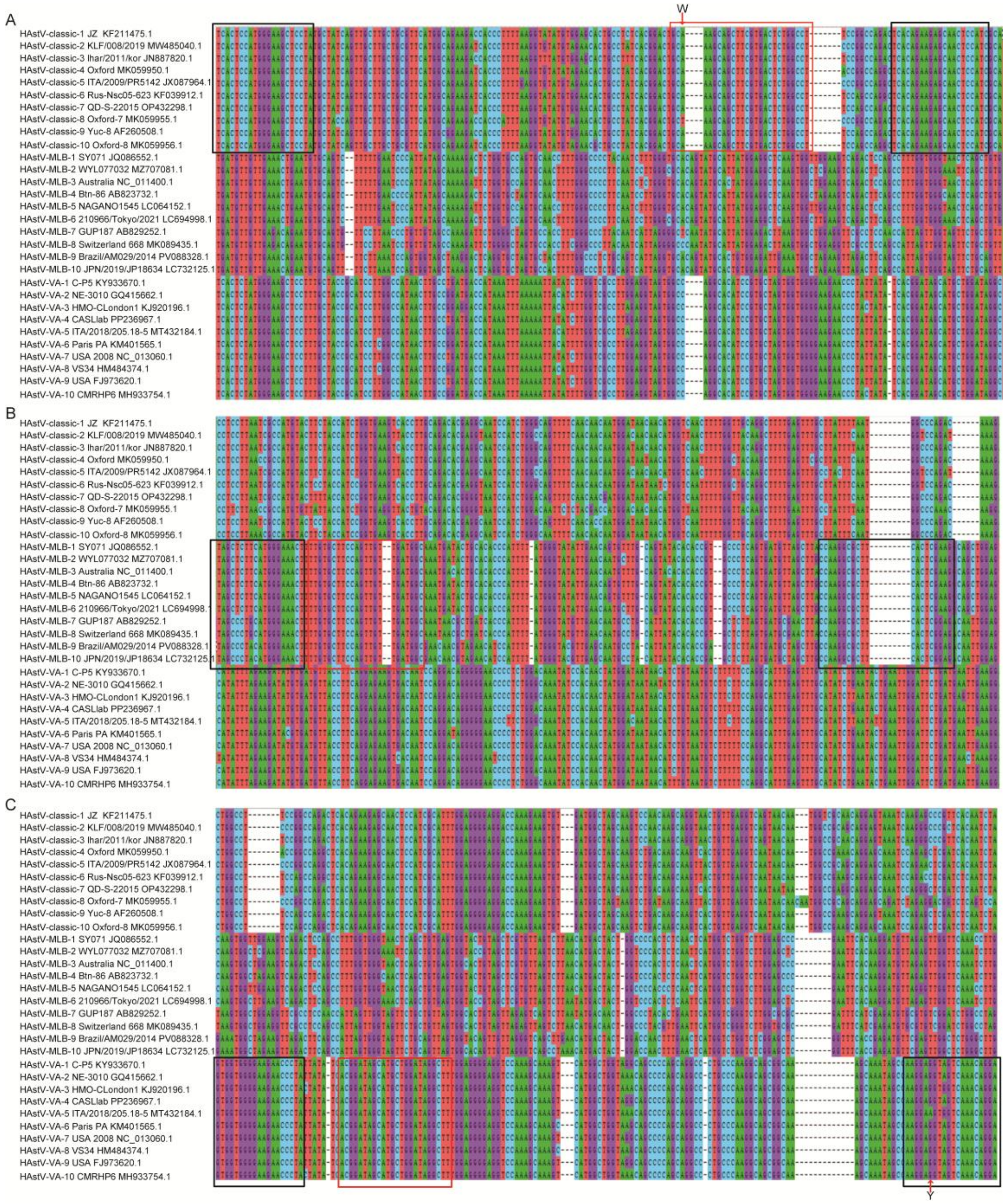

Fig. S2. Sensitivity of the conventional PCR for detecting: (A) the classical HAstV, (B) HAstV-MLB, and (C) HAstV-VA. Sensitivity tests were conducted using a 10-fold dilution series of recombinant plasmids, ranging from 10^8–10^0 copies/μL, as templates. The nuclease-free water served as the negative control.

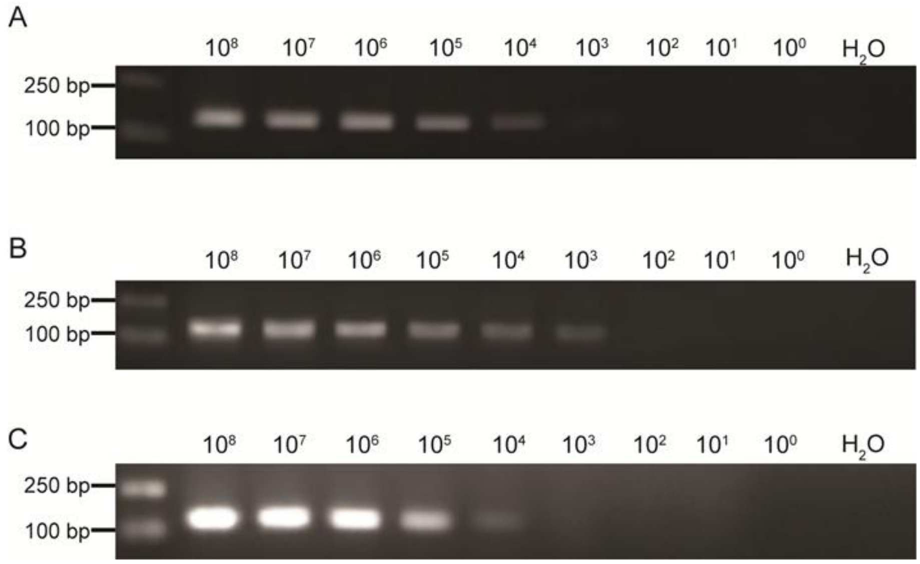

Table S1. Comparison of multiplex RT-qPCR and conventional PCR for detecting clinical specimens. "+", positive; "–", negative; "N/A", No amplication.

Sample No.	Result of Pathogen Detection					
	Multiple RT-qPCR assay			Conventional PCR		
	Classical HAstV (Cq Value)	HAstV-MLB (Cq Value)	HAstV-VA (Cq Value)	Classical HAstV	HAstV-MLB	HAstV-VA
6	19.257±0.442	N/A	N/A	+	–	–
9	27.282±0.211	N/A	N/A	+	–	–
17	31.007±0.324	N/A	N/A	–	–	–
20	26.910±0.205	N/A	N/A	+	–	–
39	24.568±0.230	N/A	N/A	+	–	–
43	26.268±0.308	N/A	N/A	+	–	–
56	23.399±0.301	N/A	N/A	+	–	–
78	26.736±0.355	N/A	N/A	+	–	–
80	22.608±0.230	N/A	N/A	+	–	–
103	25.405±0.250	N/A	N/A	+	–	–
120	25.283±0.257	N/A	N/A	+	–	–
151	26.254±0.158	N/A	N/A	+	–	–
175	23.264±0.223	N/A	N/A	+	–	–
177	20.672±0.368	N/A	N/A	+	–	–
215	26.334±0.295	N/A	N/A	+	–	–
239	33.210±0.228	N/A	N/A	–	–	–
254	21.557±0.291	N/A	N/A	+	–	–
278	26.318±0.072	N/A	N/A	+	–	–
299	25.512±0.359	N/A	N/A	+	–	–
28	N/A	19.405±0.21	N/A	–	+	–

		8				
35	N/A	26.317±0.181	N/A	−	+	−
61	N/A	22.442±0.349	N/A	−	+	−
162	N/A	27.068±0.278	N/A	−	+	−
183	N/A	23.537±0.344	N/A	−	+	−
138	N/A	N/A	25.470±0.227	−	−	+
77	N/A	25.313±0.232	26.743±0.344	−	+	+
